# G-quadruplexes of KSHV oriLyt play important roles in promoting lytic DNA replication

**DOI:** 10.1128/spectrum.05316-22

**Published:** 2023-10-06

**Authors:** Prerna Dabral, Timsy Uppal, Subhash C. Verma

**Affiliations:** 1 Department of Microbiology and Immunology, University of Nevada, Reno School of Medicine, Reno, Nevada, USA; 2 Vitalant Research Institute, San Francisco, California, USA; Barnard College, Columbia University, New York, New York, USA

**Keywords:** KSHV, DNA replication, origin of replication, G-quadruplex, RecQ1

## Abstract

Kaposi’s sarcoma-associated herpesvirus (KSHV) utilizes distinct origins of DNA replication (origin of replications) during the latent and lytic phases of its life cycle. Lytic DNA replication requires the participation of cellular and viral proteins to initiate replication at a specific region in the KSHV genome, oriLyt. These regions contain multiple secondary structures and binding sites for viral and cellular proteins. We discovered the presence of G-quadruplex (G4) sites in the oriLyt region. G-quadruplexes are secondary structures in nucleic acid sequences that are considered regulators of multiple biological processes, such as transcription, translation, and replication initiation. Importantly, herpesviruses have a high density of putative quadruplex sequence or formation sites in their genome, including in the regulatory regions, which control replication and transcription. The binding of RecQ1 to the oriLyt region of KSHV and its ability to unwind the G4 structures led us to speculate that G-quadruplex sites play an important role in lytic DNA replication. In this study, we confirmed the formation of stable G-quadruplexes through biochemical and biophysical assays. We further demonstrated that RecQ1, a helicase, associates with the G-quadruplex forming sites of the oriLyt. The functional significance of G4 sites and RecQ1 was confirmed through the stabilization of G4 and depletion of RecQ1 levels through shRNA. Furthermore, the detection of replication initiation through single-molecule analysis of the replicated DNA demonstrated that G4 stabilization leads to subdued replication initiation at the oriLyt. This confirmed the role of the G-quadruplex in regulating viral DNA replication, which can be used to control virus growth.

**IMPORTANCE:**

Biological processes originating from the DNA and RNA can be regulated by the secondary structures present in the stretch of nucleic acids, and the G-quadruplexes are shown to regulate transcription, translation, and replication. In this study, we identified the presence of multiple G-quadruplex sites in the region (oriLyt) of Kaposi’s sarcoma-associated herpesvirus (KSHV) DNA, which is essential for DNA replication during the lytic cycle. We demonstrated the roles of these G-quadruplexes through multiple biochemical and biophysical assays in controlling replication and efficient virus production. We demonstrated that KSHV achieves this by recruiting RecQ1 (helicase) at those G-quadruplex sites for efficient viral DNA replication. Analysis of the replicated DNA through nucleoside labeling and immunostaining showed a reduced initiation of DNA replication in cells with a pharmacologic stabilizer of G-quadruplexes. Overall, this study confirmed the role of the G-quadruplex in regulating viral DNA replication, which can be exploited for controlling viral DNA replication.

## INTRODUCTION

Kaposi’s sarcoma-associated herpesvirus (KSHV) or HHV-8, a gammaherpesvirus, is the etiological agent of Kaposi’s sarcoma, primary effusion lymphoma, multicentric Castleman’s disease, and KSHV inflammatory syndrome (1 – 4). Like other viruses of the Herpesviridae family, the life cycle of KSHV is biphasic, characterized by a default latent phase and a short and transient lytic phase. The latent phase of the viral life cycle is characterized by restricted gene expression and replication of the viral genome. The latent DNA replication occurs once per cycle, facilitated by the latent associated nuclear antigen (LANA) after recruiting the cellular replication machinery to the origin of latent DNA replication (5). However, the lytic phase of infection involves an orchestrated expression of immediate early (IE), early (E), and late (L) genes with the replication of the viral genome to produce infectious virions following packaging into capsids and teguments (6). Lytic replication initiates at the origin of lytic DNA replication (oriLyt), during which the virus uses viral and cellular proteins to replicate its genome, presumably through a rolling circle mechanism. Two copies of oriLyt in the KSHV genome, oriLyt-L and oriLyt-R, are present between the open reading frames (ORFs) K4.2 and K5, and between K12 and ORF71, respectively (7, 8). The oriLyt regions are 1.7 Kb in length, consisting of a highly similar 1.1 Kb long core sequence along with 600 bp GC repeats, present in the form of 20 bp and 30 bp tandem repeats. The core sequence contains C/EBP motifs, which are required for lytic DNA replication through its binding to the K8 protein at the AT palindromic sequence. In addition, the core sequence contains an RTA-dependent promoter, which includes RTA binding site, RRE (RTA response element), and TATA box consensus sequence (9, 10). Proteins that are essential for lytic DNA replication are conserved among herpesviruses forming the core replication machinery, consisting of ORF9 (DNA polymerase), ORF6 (single-stranded binding protein), ORF59 (processivity factor), ORF44 (helicase), PRF56 (primase), and ORF40/41 (primase-associated factor) (11 – 13). ORF50 or RTA and K8 are essential for lytic DNA replication and have been shown to form the pre-replication complex with the core proteins following recruitment to the oriLyt region (9, 14).

In addition to the viral proteins, many cellular proteins were identified to bind to the oriLyt region, including (Topo) I and II, MSH2/6, RecQL, DNA-PK, poly (ADP-ribose) polymerase 1 (PARP-1), Ku autoantigens, and SAF-A (scaffold attachment factor A). These proteins are involved in several cellular processes, such as replication, recombination, and repair, and are shown to be present on the viral replication foci during lytic replication.

G-quadruplexes or G4 structures are thermodynamically stable non-canonical structures formed in DNA/RNA sequences carrying four runs of at least three guanines separated by a few bases (15). The building block of G-quadruplexes is a guanine tetrad, formed by Hoogsteen hydrogen bonding between four guanines giving rise to a planar structure that stacks on top of each other through π-π interactions. The presence of monovalent cations such as K+ ions and Na+ ions reduces repulsion between negatively charged oxygen atoms, thereby promoting the formation of G-quadruplexes (16). These structures have been identified in eukaryotes, prokaryotes, and viruses and are implicated in regulating various biological processes, including replication, transcription, and translation (17). The presence of G-quadruplexes has been reported in several human viruses, including human immunodeficiency virus (HIV), hepatitis B virus, hepatitis C virus, Ebola virus, Zika virus, Epstein-Barr virus (EBV), and KSHV (18, 19).

Recently, G-quadruplexes became important regulators of replication initiation at the origin sites (20). These structures have been identified in approximately 90% of the replication origins in humans and mice, and about 70% of the origins/replication initiation sites in Drosophila are reported to be a few hundred base pairs downstream of G4 sites (21). G-quadruplex regions have been identified as the potential site of replication initiation, and their roles are demonstrated in the early replication (22, 23). Moreover, the association of G-quadruplexes with proteins involved in unwinding or resolving negatively supercoiled duplex DNA has led to the hypothesis that the unfolding of these secondary structures located in origin facilitates the recruitment of many replication factors such as ORCs, which have affinities for these G4 sites (22, 24).

Moreover, the presence of G-quadruplexes in the genome could be detrimental, as they serve as roadblocks to the movement of cellular enzymes involved in replication or transcription. Therefore, cells employ ways to resolve these structures for the precise functioning of biological processes. Helicases are the cellular motor proteins that separate two annealed strands of nucleic acids using energy derived from the nucleoside triphosphate hydrolysis (25 – 28). Their role in unwinding G4s was initially reported in the late 1990s, with SV-40 helicase, Bloom syndrome helicase, *Saccharomyces cerevisiae* proteins, Sgs1p helicase, and SEP1 protein reported to resolve the G4 structures (29 – 32). RecQ belongs to a conserved family of DNA helicases called guardians of the genome due to their roles in DNA replication, recombination, and repair (33, 34). The human RecQ helicases include RECQ1, WRN, BLM, RECQ4, and RECQ5 and Bloom, Werner, and Rothmund-Thompson syndrome, which is associated with mutations in BLM, WRN, and RecQ4, respectively (35). BLM helicase encoded by the BLM gene has been shown to unwind the G4 structures, and its G4 unwinding activity has been implicated in promoting telomere replication and genome integrity (36 – 39). Another member of the RecQ family, WRN helicase, has been shown to reduce genome instability due to its ability to resolve (CGG) repeats associated with fragile X syndrome in addition to promoting efficient replication at the telomeric sequences (40 – 42). Recent studies showed that RecQL4, a member of the RecQ family important for replication initiation and fork progression, binds to the G4 structures (43, 44).

RecQ1, another member of the RecQ family, is the most abundant RecQ protein in the cells and gets upregulated in EBV-transformed B cells (45, 46). RecQ1 has been shown to repress chromosome instability, reduce DNA damage, and promote initiation at the paused replication forks (47 – 49). In addition to being linked to replication initiation at the origin, RECQ1 binds to the oriLyt region of KSHV and EBV (50, 51). We previously reported G-quadruplexes’ formation throughout the genome and its impact on the latent DNA replication (52). In this study, we demonstrate the formation of these secondary structures in the oriLyt region through biophysical and biochemical assays.

Moreover, we determined the binding of RecQ1 to the G4 forming region of the oriLyt and substantiated the indispensable role of RecQ1 in KSHV lytic replication through the depletion of RecQ1 and stabilization of G-quadruplexes. Additionally, we evaluated the relevance of G-quadruplex formation in lytic replication through the ablation of G4 sites by site-direction mutagenesis. We also used ligands to stabilize the formation of these structures to determine their effect on the initiation of lytic replication on individual DNA molecules.

## MATERIALS AND METHODS

### Cell lines, plasmids, and antibodies

The KSHV-positive BCBL-1/BC3 cell lines were grown in RPMI 1640 supplemented with 10% fetal bovine serum (FBS), 2 mM glutamine, 5 U/mL penicillin, and 5 ug/mL streptomycin. BJAB-L-YFP cell line was grown in RPMI 1640 supplemented with 10% FBS, 2 mM glutamine, 5 U/mL penicillin and 5 ug/mL streptomycin, and 0.5 µg/mL puromycin. iSLKTet-RTA-Bac16-wild-type (wt) and iSLKTet-RTA-Bac16-RTA-STOP cells were maintained in DMEM supplemented with 10% tet-free FBS with additional 600 µg/mL hygromycin B, 400 µg/mL G418, and 1 µg/mL puromycin. iSLK.219 cells with recombinant KSHV BACs were induced by doxycycline. The following commercial antibodies were used for this study: rabbit anti-RecQ1 (Bethyl Laboratories, Inc.), mouse anti-GAPDH (US Biological), and rabbit anti-RTA (custom synthesized at GenScript, Inc.). Site-directed mutagenesis was performed in wt 8088sc (8088wt) plasmid carrying the KSHV oriLyt-L sequence using QuikChange Lightning Site-Directed mutagenesis kit, according to the manufacturer’s protocol. The integrity of the resulting mutated plasmid (i.e., 8088mut) was confirmed by sequencing at the Nevada Genomics Center, University of Nevada, Reno, NV, USA.

### CD spectroscopy

CD spectroscopy was performed on wt-oriLyt (5′-CACG**GGG**TTGTTCGGTGGC**GGG**G**GGGGGG**CTAG-3′) and scrambled oligos (5′-ACTATATTGTTCAATAACTATATTATAAATACT-3′) using Aviv Biomedical spectrometer, where circularly polarized UV light was used to record CD spectra in a series of progressive scans from 320 nm to 200 nm in a quartz cuvette of 1 mm path length at 25°C. The wt- and scrambled oriLyt DNA oligonucleotides were used at a final concentration of 5 µM in sodium cacodylate buffer (10 mM, pH 7.4) with 100 mM KCl. G-quadruplexes were allowed to form in the oligos by initial denaturation at 95°C for 5 min, followed by slow cooling at room temperature.

### Electrophoretic mobility shift assay

Electrophoretic mobility shift assay (EMSA) was performed as described before (52). Briefly, wt-oriLyt and scrambled oriLyt DNA oligonucleotides were labeled with radioactive ^32^P using Terminal deoxynucleotide transferase, TdT (New England Biolabs), and resuspended at a final concentration of 2 µM in 10 mM sodium cacodylate buffer with 100 mM KCl. Following denaturation and slow cooling, the oligos were resolved on a 15% native polyacrylamide gel in 1× TBE buffer with 100 mM KCl, gels were dried using Gelair gel dryer (Bio-Rad Inc.), and autoradiography was performed using a phosphorimager (GE Healthcare Life Sciences). For G4 disruption, antisense oligos were incubated with the labeled oligos before denaturation.

### *In vitro* DNA pull-down assay

The wt-oriLyt oligo (5′-CACG**GGG**TTGTTCGGTGGC**GGG**G**GGGGGG**CTAG-3′) and scrambled oligo (5′-ACTATATTGTTCAATAACTATATTATAAATACT-3′) were biotinylated at the 3′end Terminal deoxynucleotide transferase, TdT (New England Biolabs). The cellular lysate was prepared from approximately 20 million KSHV-positive BCBL-1 cells, which were lytically induced for 24 hours using sodium butyrate (NaB) and 12-o-tetradecanoylphorbol-13-acetate (TPA), harvested and lysed in 1% NP-40 lysis buffer with protease inhibitors. The samples were sonicated and centrifuged at 4°C to remove cellular debris. The purified biotinylated DNA oligos were incubated with the lysate for 3 hours at 4°C, and Pierce Streptavidin agarose beads (Thermo Fisher Scientific) were added to the samples for 2 hours to pull down the proteins bound to the biotinylated DNA. The beads were washed thrice with 1% NP-40 lysis buffer, following which they were loaded onto a 9% SDS-PAGE, transferred to a nitrocellulose membrane, and probed with RecQ1 antibody.

### Chromatin immunoprecipitation assay (ChIP)

Chromatin immunoprecipitation was performed using the iDeal ChIP qPCR kit (Diagenode, Inc.) according to the manufacturer’s instructions. Briefly, KSHV-positive cells were induced for lytic reactivation for 24 hours, following which approximately 8 million cells were fixed with 1% formaldehyde for 10 min at room temperature, and crosslinking was stopped by the addition of glycine at a final concentration of 125 mM for 5 min. The cells were washed two times with ice-cold phosphate-buffered saline (PBS), and nuclei were extracted following cell lysis using kit-supplied buffers. Cells were finally lysed in chromatin shearing buffer supplemented with protease inhibitors for 10 min on ice, and chromatin was sonicated using a Bioruptor (Diagenode) to an average length of 200 to 300 bp. The lysates were centrifuged at 16,000 *g* for 10 min to remove the cell debris, and the supernatant was incubated with specific antibodies and magnetic beads overnight at 4°C. The following day, the beads were collected and subsequently washed at 4°C for 5 min each with wash buffers iW1, iW2, iW3, and iW4. The beads-bound DNA was subjected to proteinase K digestion, and the DNA was finally eluted from the beads by incubation at 100°C for 15 min. Purified ChIP DNA samples and the inputs were subjected to amplification with specific primers using ABI StepOne Plus, a real-time PCR machine (Applied Biosystems). The binding of protein to chromatin was analyzed as a percentage of the input as well as the fold enrichment method compared to the control through the ∆∆Ct method.

### Transfection of oriLyt plasmids and transient replication assay

Approximately 20 million BCBL-1 cells were transfected with 30  µg of oriLyt plasmids (either 8088wt or 8088mut) using the Neon transfection system (Thermo Fisher Scientific). The cells were allowed to recover for 24 hours and induced lytic reactivation (24 hours for chromatin immunoprecipitation assay and 48 hours for replication assay). For replication assay, cells were washed with PBS, followed by DNA extraction using a modified Hirt’s lysis method, described earlier (53, 54). A fraction of DNA was linearized with EcoRI and the remainder with DpnI and EcoRI to remove the non-replicated DNA. Digested DNA was resolved, transferred to a nylon membrane, and then hybridized with ^32^P-labeled 8088sc probes. The auto-radiographic signals were detected using PhosphorImager, according to the manufacturer’s instructions (Molecular Dynamics, Inc.).

### Single-molecule analysis of the replicated DNA (SMARD)

SMARD was used to analyze the effect of G-quadruplex formation on the initiation of lytic DNA replication as described previously (55, 56). BCBL-1 cells were treated with G4 stabilizing compound TMPyP4 at a concentration of 10 µM and induced lytically for 24 hours. The cells were sequentially labeled with 30 µM 5-iodo-2′-deoxyuridine (IdU) (Sigma-Aldrich) at 37°C for 4 hours, washed with PBS, and labeled with second nucleotide analog, 5′-chloro-2′-deoxyuridine (CldU) at 30 µM (Sigma-Aldrich) for 4 hours. The cells were finally washed and resuspended in PBS (1 × 10^6^ cells per mL) and molten 1% InCert agarose (Lonza Rockland, Inc., Rockland, ME, USA) in PBS (1:1 vol/vol). Agarose gel plugs embedded with labeled cells were made by solidification in a cold plastic mold on ice for 30 min. The plugs were incubated in lysis buffer (1% *n*-lauroylsarcosine, 0.5 M EDTA, and 20 mg/mL proteinase K) for at least 72 hours at 50°C, following which the proteinase K was removed by washing the plugs with 1X TE and phenylmethanesulfonyl fluoride (PMSF) (Sigma-Aldrich). Next, the KSHV genome was linearized using *PmeI* (New England Biolabs Inc.), where the plugs were first washed with pre-digestion buffer (10 mM MgCl_2_ and 10 mM Tris-HCl, pH 8.0) before digesting with 70 units of *PmeI* at 37°C overnight. The *PmeI* digested gel plugs were rinsed twice with TE, cast onto a 0.7% SeaPlaque GTG agarose gel (Lonza Rockland, Inc.), and resolved by PFGE for 36 hours. KSHV genome was detected using Southern blotting and hybridization with a ^32^P-labeled KSHV TR-specific probe. Band specific for KSHV genome was excised from the gel, and DNA was extracted using GELase treatment (Epicentre Biotechnologies, Madison, WI, USA, 1 unit per 50 µL of agarose suspension) that digests the agarose releasing the DNA into suspension. The DNA was then stretched on 3-aminopropyltriethoxysilane (Sigma-Aldrich) coated glass slides and denatured in alkaline denaturing buffer (0.1 N NaOH/70% ethanol, and 0.1% ß-mercaptoethanol) and fixed with 0.5% glutaraldehyde. The DNA was hybridized overnight with biotinylated probes, and the next day, slides were rinsed in 2× SSC (1× SSC) 1% SDS, washed in 40% formamide solution containing 2× SSC at 45°C for 5 min, and rinsed in 2× SSC-0.1% IGEPAL CA-630. The slides were washed four times with 4× SSC-0.1% IGEPAL CA-630, followed by blocking with 1% BSA for 20 min. Next, the slides were treated with NeutrAvidin Alexa Fluor 350 (Life Technologies Inc.) and biotinylated anti-avidin antibodies (Vector Laboratories, Inc.) for 20 min each, after washing with PBS containing 0.03% IGEPAL CA-630 in between. Slides were further treated with NeutrAvidin Alexa Fluor 350 for 20 min and washed as mentioned above. Following this, the slides were incubated with mouse anti-IdU monoclonal antibody, mouse anti-CldU monoclonal antibody, and biotinylated anti-avidin D for 1 hour. After washing, the slides were incubated with NeutrAvidin Alexa Fluor 350 and fluorescent secondary antibodies against mouse and rabbit, i.e., Alexa Fluor 488 and Alexa Fluor 594 (Invitrogen Molecular Probes), for 1 hour. The slides were washed again, and coverslips were mounted with ProLong gold anti-fade reagent (Life Technologies Inc.), followed by fluorescence microscopy.

### RecQ1 knockdown using lentiviral vectors

The pTRIPZ lentiviral vector (Dharmacon, GE Life Sciences) containing shRNA for RecQ1 was co-transfected with lentivirus packaging vectors, pCMV-dR8.2 and pCMV-VSVG (Addgene, Inc.) into Lenti-X 293T (Takara Bio) cells using polyethyleneimine (PEI) (Polysciences, Inc.) to generate the lentiviral particles. Lentivirus containing supernatants from the transfected cells were collected for 5 days, followed by concentration of the virus by ultracentrifugation (25,000 rpm, 1.5 hours, 4°C). The concentrated virus was used for transducing the target cells, BCBL-1, in the presence of 5 µg/mL polybrene, followed by selection with 1  µg/mL puromycin. The cells were treated with 1 µg/mL doxycycline for at least 72 hours for the induction of knockdown. The RNA interference (RNAi) efficiency was assessed by western blot analysis using RecQ1 antibody.

### IdU labeling and immunoprecipitation of replicated DNA

IdU labeling was performed as described previously (54). Briefly, cells were pulsed with 30  µM of IdU for 30 min, washed twice with cold PBS, and the episomal DNA was extracted by a modified Hirt’s method. The samples were dissolved in 500 µL TE (10  mM Tris-HCl, 1  mM EDTA), sonicated to get an average length of 700  bp, and heat denatured at 95°C for 5  min. Ten percent of the extracted DNA was used as input control, and 1  µg of the mouse of anti-IdU antibody was added to samples and incubated at room temperature with constant rotation for 1  hour. Magnetic Protein A/G Antibody was used to pull down the bound IdU labeled DNA, and the beads were washed once with 1× IP buffer (10 mm NaPO4 pH 7.0, 140 mM NaCl, and 0.05% Triton X-100), resuspended in 200  µL of lysis buffer [50  mM Tris-HCl (pH 8.0), 10   mM EDTA, 0.5% SDS, 0.25  mg/mL proteinase K] and incubated overnight at 37°C for elution. This was followed by adding 100 µL of lysis buffer and incubating at 50°C for 1 hour. The eluted DNA was phenolized and precipitated for the quantitation of IdU-labeled DNA in a real-time PCR by amplifying the oriLyt region. The assay was done with three biological replicates, and the fold change was calculated by the ∆∆Ct method.

### KSHV virion purification

KSHV virions were purified as described previously (57). Briefly, 15 million cells were induced with 0.3M NaB and 20 ng/mL TPA for 96 hours; culture supernatant was cleared by centrifugation and filtered through a 0.45-µm filter to remove cell debris. The virus was concentrated by ultracentrifugation (25,000 rpm for 2 hours at 4°C), resuspended in serum-free RPMI, and 50 µL of virus supernatant diluted with 250 µL of 1× PBS was digested with DNase-I (5 U/50 µL of supernatant) at 37°C for 1 hour. DNase-I was heat inactivated at 70°C for 10 min, and supernatants were mixed with an equal volume of lysis buffer (0.1 mg/mL of proteinase K in water) and incubated at 50°C for 1 hour. Proteinase K was heat-inactivated at 75°C for 20 min, and DNA was purified using PCI (Phenol: Chloroform: Isoamyl alcohol), precipitated with ethanol at −20°C and resuspended in sterile Milli-Q water. The viral DNA was amplified with qPCR using primers specific for LANA, and virus quantities were calculated based on the LANA standards. The assay was done with three biological replicates.

### Quantitation of KSHV RNA

Total RNA was isolated from the cells using Illustra RNAspin Mini Kit (GE Healthcare) according to the manufacturer’s protocol. cDNAs were generated using a high-capacity RNA-to-cDNA kit (Applied Biosystems Inc.) per the manufacturer’s protocol and quantified using specific primers. The Ct values were normalized to the housekeeping gene, GAPDH. The assay was done with three biological replicates, and the fold change was calculated by the ∆∆Ct method.

### Determination of viral genome copies

Five million KSHV-positive cells were induced for lytic reactivation for 24 hours with or without compound treatment. Total genomic DNA was isolated using PureLink Genomic DNA Purification Kit according to the manufacturer’s instructions and quantified using primers specific to the DNA sequence of RTA. The Ct values were normalized to the housekeeping gene, GAPDH. The assay was done with three biological replicates, and the fold change was calculated by the ∆∆Ct method.

### Statistical analysis

All the assays were performed in triplicate, and *P*-values were calculated by a two-tailed *t*-test using GraphPad Inc. (Prism 8) software for statistical significance. Asterisks represent the *P*-value <0.05 (*), *P*-value <0.01 (**), and *P*-value <0.001 (***), where ns denotes non-significant.

## RESULTS

### Sequences of oriLyt region formed stable G4 structures

**Fig 1:**
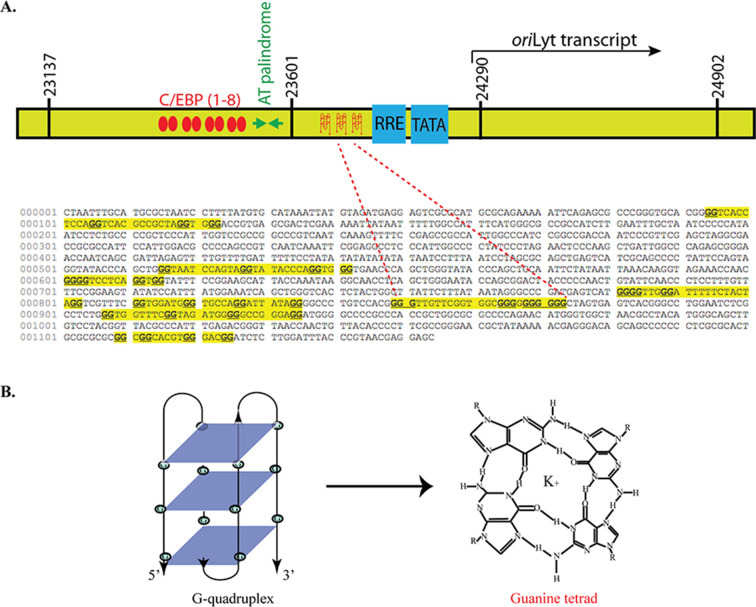
oriLyt region formed stable DNA G-quadruplexes. (**A**) A schematic of the oriLyt region showing different domains, with the G-quadruplex (**G4**) forming regions highlighted in yellow. (A) The oriLyt sequence was imported into QGRS mapper software, which predicts G-quadruplex formation as a function of G-score. (B) A guanine tetrad shows Hoogsteen hydrogen bonding between four guanine residues stabilized by a potassium ion in the center. Three guanine tetrads stack on top of each other to form a G-quadruplex.

**Fig 2:**
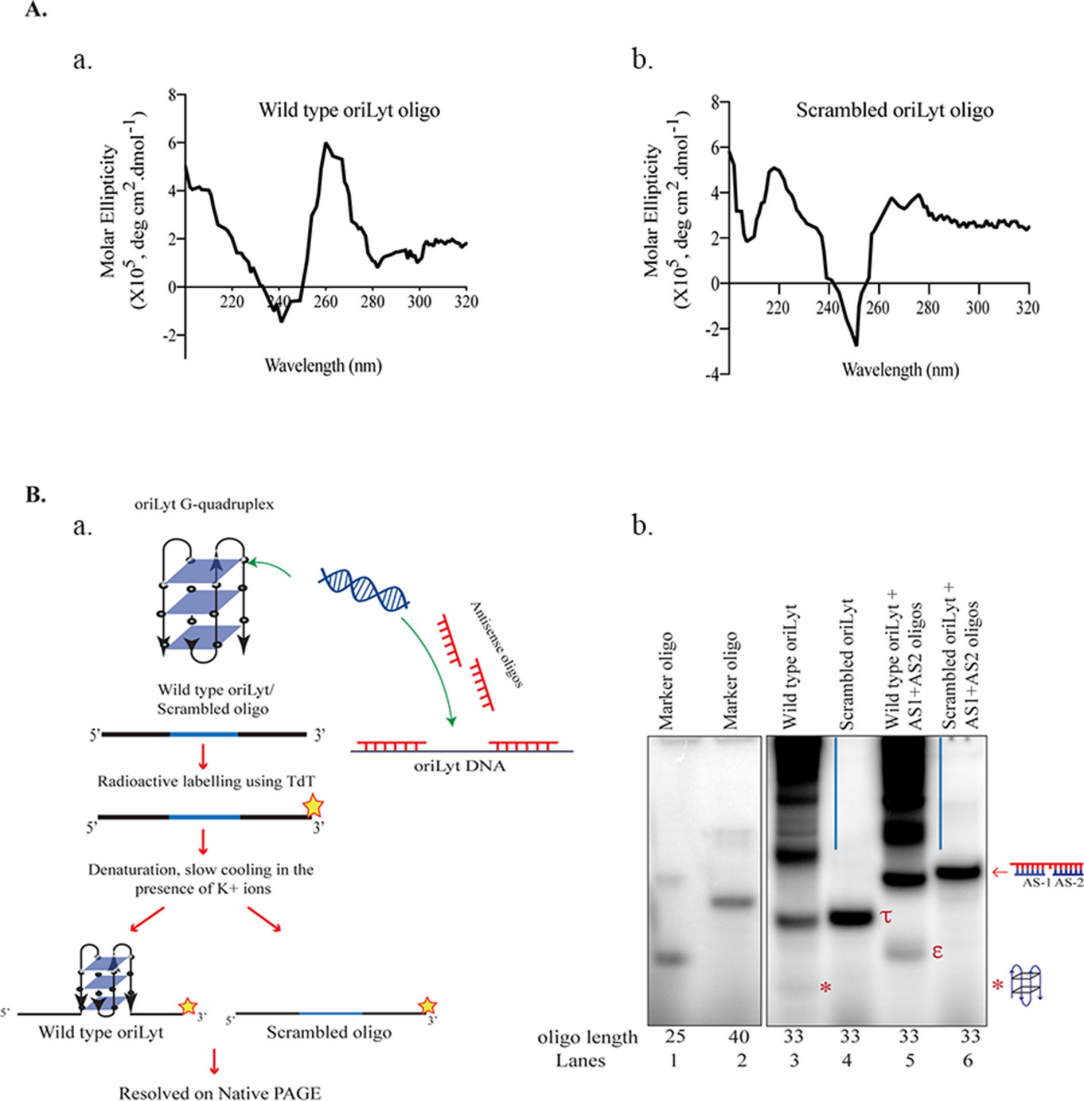
Biophysical and biochemical analysis confirmed the presence of G-quadruplexes in the oriLyt region. (**A**) Circular dichroism spectral analysis of (**a**) oriLyt wild-type oligonucleotide having a high G-score and (**b**) scrambled DNA oligonucleotide which could not form G4s was used as a control. The oligos were scanned for a wavelength range of 320 nm to 200 nm, and molar ellipticity was plotted on the Y-axis with a wavelength on the X-axis. (**B**) (**a**) Schematic of steps involved in Native PAGE analysis of wild-type oriLyt or scrambled oriLyt. (**b**) Electrophoretic mobility shift assay (EMSA) was performed in the presence of K+ ions on wild-type oriLyt and scrambled oligos labeled with ^32^P and resolved on a native polyacrylamide gel. Antisense oligos (AS1 and AS2) complementary to wild-type oriLyt oligo, added in molar excess, were used in the indicated lanes to confirm that the G-quadruplex forming sequence caused the mobility shift. Marker oligos of lengths 25nt and 40nt are in lanes 1 and 2, respectively. Wild-type and scrambled oligo (33nt) of oriLyt are in lanes 3 and 4. G4 forming band is represented by an asterisk. The band formed by the scrambled oligo is represented by tau (τ). Shift in the band mobility of G4 oligo due to the antisense oligo is indicated by (ε), lane 5. Lane 6, scrambled oligo with antisense oligos. Vertical lines in lanes 3 and 5 represent the high molecular weight forms of the G-quadruplexes.

DNA G-quadruplexes are secondary structures formed in G-rich stretches of DNA that have been implicated in several biological processes. These structures have been reported in the latent nuclear protein of EBV, terminal repeat region of KSHV, viral gene promoters, and in the oriLyt region of the human cytomegalovirus (HCMV) (52, 58, 59). Given the critical role of the G4 structures in the viral life cycle, we were interested in investigating their role in the lytic DNA replication of KSHV. First, we analyzed the oriLyt sequence of KSHV for the presence of G-quadruplex using a web-based tool, QGRS mapper, that predicts the formation of these structures (http://bioinformatics.ramapo.edu/QGRS/index.php) (60). G-scores, a predictor of the G-quadruplexes formation, were determined for the oriLyt region of KSHV, and the region with the high G-scores is represented by a yellow highlighted region (Fig. 1A). The oriLyt sequence with the highest G-score was located between the AT-rich region and the RRE element. Since G4 structures are unique concerning their structure and folding (Fig. 1B), they display distinct biophysical and biochemical properties. We validated the formation of G-quadruplexes by performing CD spectroscopy of an oligo containing the wild-type oriLyt G4 site or a scrambled oligo. Oligo with specific G4 site showed a spectral pattern with a maximum at 260 nm and a negative minimum at 240 nm for wild-type oriLyt oligo (Fig. 2A, panel a). This pattern is specific for G-quadruplexes and was not observed for scrambled oligo (Fig. 2A, panel b). We further confirmed the G-quadruplex formation on the oriLyt oligo in an electrophoretic mobility shift assay. The wild-type and scrambled oriLyt DNA oligos were labeled with ^32^P using Terminal deoxynucleotidyl transferase and resolved in the presence of K+ ions on a 15% native gel, following which autoradiography was used to determine the mobility of these oligos (Fig. 2B, panel a). Our results showed that the wild-type oriLyt G4-forming DNA oligo migrated faster than the scrambled oligo, possibly due to the formation of condensed G-quadruplex structures (Fig. 2B, panel b). To corroborate that the increased mobility of the wild-type oriLyt oligo was caused by the formation of these secondary structures, we used antisense oligos to disrupt the formation of G-quadruplexes. The antisense oligos (AS1: GCCACCGAACAACCCC and AS2: CACTAGCCCCCCCC) were complementary to the G-rich region of wild-type oriLyt oligo. Following the incubation of these antisense oligos with G4 oriLyt oligo and resolution on a native gel, we observed a shift in the mobility of wild-type oriLyt oligo in the presence of specific antisense oligos as compared to the wild-type oriLyt oligo without the anti-sense oligos (Fig. 2B, panel b, compare lane 3 with lane 1). Based on these findings, we concluded that the fast-migrating band of the oriLyt G4 oligos on the native gel is due to the formation of G-quadruplexes as the disruption of these structures by antisense oligos led to a significant shift in the mobility of these oligos. These results validated that the regions of the oriLyt can form G-quadruplexes.

### RecQ1 bound to the region oriLyt with G-quadruplex

**Fig 3:**
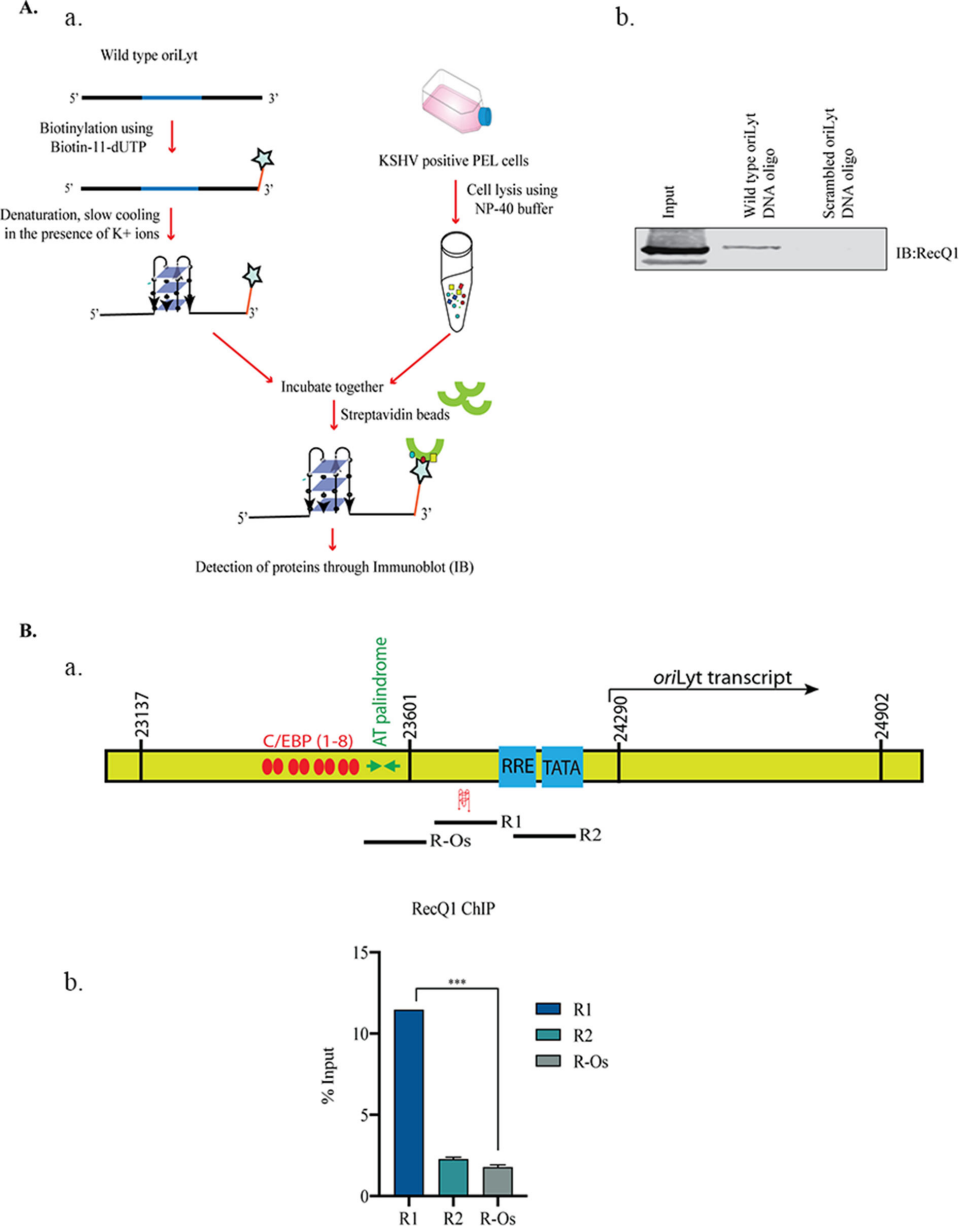
RecQ1 bound to the G-quadruplex forming site of the oriLyt. (**A**) Schematic of the steps involved in DNA affinity pull-down assay. (**b**) Immunoblot showing RecQ1 binding to the wild-type oriLyt. Wild-type oriLyt and scrambled oligo (negative control) were biotinylated and incubated with cell lysate from lytically induced KSHV positive BCBL-1 cells, and streptavidin beads were added to pull down DNA-bound proteins followed by resolution of the samples on SDS-PAGE gel and detection by RecQ1 antibody. (**B**) (**a**) Schematic of the oriLyt region depicting different regions (R1, R2, and R-Os) used for PCR amplification. (**b**) BCBL-1 cells were induced for lytic reactivation for 36 hours, following which the cells were harvested, crosslinked, and utilized to perform ChIP assay with RecQ1 antibody to determine the relative binding of RecQ1 at different regions (R1, R2, and R-Os) of oriLyt using specific primers.

The G-quadruplexes play a major role in multiple cellular processes through their interaction with a variety of cellular proteins, which either support the formation or destabilize these structures (17). Interestingly, an earlier study identified the association of RecQ1, a cellular helicase, with the oriLyt region, possibly through its interaction with other viral proteins (50). RecQ proteins have been shown to facilitate DNA replication through their G4-resolving ability (61). This prompted us to investigate the binding ability of G4s to RecQ1 with respect to oriLyt DNA. To achieve this, we performed a DNA affinity pull-down assay with wild-type oriLyt oligo carrying G4 sites or a control oligo with scrambled G4 sites, biotinylated at the 3′ end (Biotin-11-UTP), and incubating with the cellular extract from lytically reactivated KSHV positive BCBL-1 cells. After the incubation of the biotinylated oligos with the cellular lysates, G4 associating proteins were captured with the streptavidin beads, following stringent washes to remove any non-specifically bound proteins (Fig. 3A, panel a). The bound proteins were resolved on an SDS-PAGE for the detection of the protein of interest through immunoblotting. An immunoblot with an anti-RecQ1 antibody showed a specific pull-down of RecQ1 with wild-type oriLyt oligo but not with the scrambled oligo (Fig. 3A, panel b). Next, we wanted to validate our findings *in vivo* by performing a chromatin immunoprecipitation assay using RecQ1 antibody on BCBL-1 cells induced for lytic reactivation. Three sets of primers covering the regions of oriLyt were used for quantifying a relative binding/enrichment of RecQ1 throughout the oriLyt. These oriLyt regions with primer sets included: R1, a region with a G4 site; R2, a region that has an RTA response element (RRE) but not a G4 site; and R-Os, a region outside the RecQ1-binding area of oriLyt (50) (Fig. 3B, panel a). Relative enrichment of these regions in the chromatin-bound to RecQ1 showed a specific association of RecQ1 with the R1 region more than R2 or the R-Os regions. Collectively, these findings revealed that RecQ1 associate with the region of the oriLyt containing a G4 site (Fig. 3B, panel b).

### RTA recruited RecQ1 to the oriLyt region

**Fig 4:**
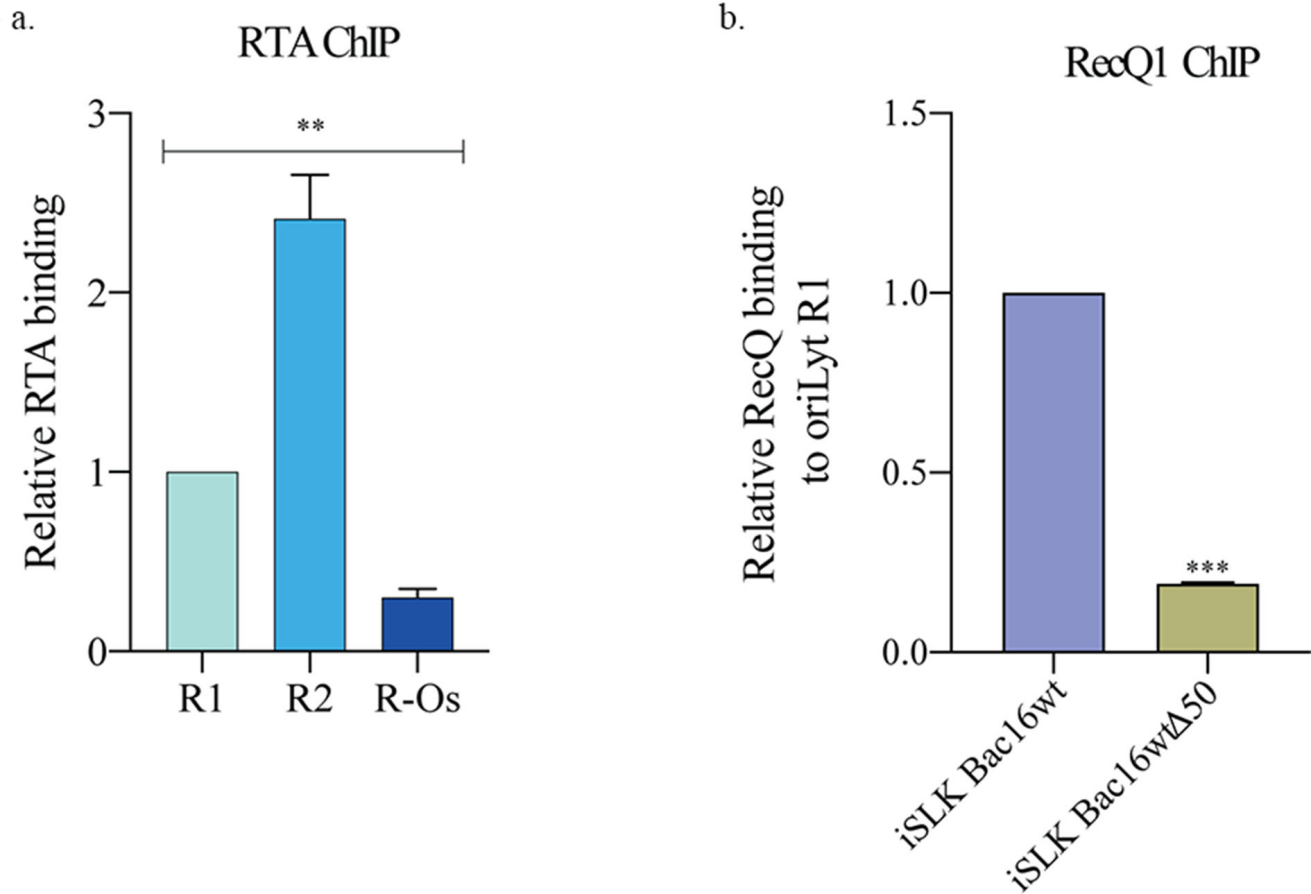
RTA facilitated the binding of RecQ1 at oriLyt. (a) ChIP assay performed using lytically induced BCBL-1 cells, where cells were harvested, crosslinked, and chromatin-bound DNA was pulled down using RTA antibody to determine the relative enrichment of RTA at different regions of oriLyt (R1, R2, and R-Os) using specific primers. (**b**) ChIP assay was performed using lytically induced iSLK-Tet-RTABAC16-WT and iSLKTet-RTA-Bac16-RTASTOP cells where the cells were harvested, crosslinked, and chromatin-bound DNA was pulled down using RecQ1 antibody to determine the relative binding of RecQ1 on oriLyt using primers specific for R1.

RTA plays an indispensable role in lytic DNA replication through its interaction with other viral and cellular proteins. RTA has been shown to bind to RRE in the oriLyt region, activate the promoter of oriLyt, and initiate transcription. In addition, it has been shown to interact with proteins of the pre-replication complex and it recruits them to the origin to facilitate lytic DNA replication (14). A previous study showed that RecQ1 binds to RTA directly, and its binding to the oriLyt region is compromised in the absence of the RRE domain (50). In this report, we further investigated the binding of RTA and RecQ1 with respect to G4 forming sites in the oriLyt. To achieve this, we first performed a chromatin immunoprecipitation assay with an anti-RTA antibody on lytically induced BCBL-1 cells and quantified the enrichment of RTA on the regions of oriLyt described above. Not surprisingly, we found a specific enrichment of RTA onto the R2 region, which contains the RRE domain but not a G4 site (Fig. 4a). We further asked whether the enrichment of RecQ1 was dependent on the presence of RTA at the RRE site of oriLyt; we analyzed the enrichment of RecQ1 in cells lacking RTA expression. Chromatin immunoprecipitation with anti-RecQ1 antibody from the iSLKTet-RTABAC16-WT and iSLKTet-RTA-Bac16-RTASTOP cells (without RTA), we observed a relatively reduced binding of RecQ1 at R1 region in cells without RTA compared to wild-type cells (Fig. 4b).

### Stabilization of G4 sites and depletion of RecQ1 inhibited lytic DNA replication

**Fig 5:**
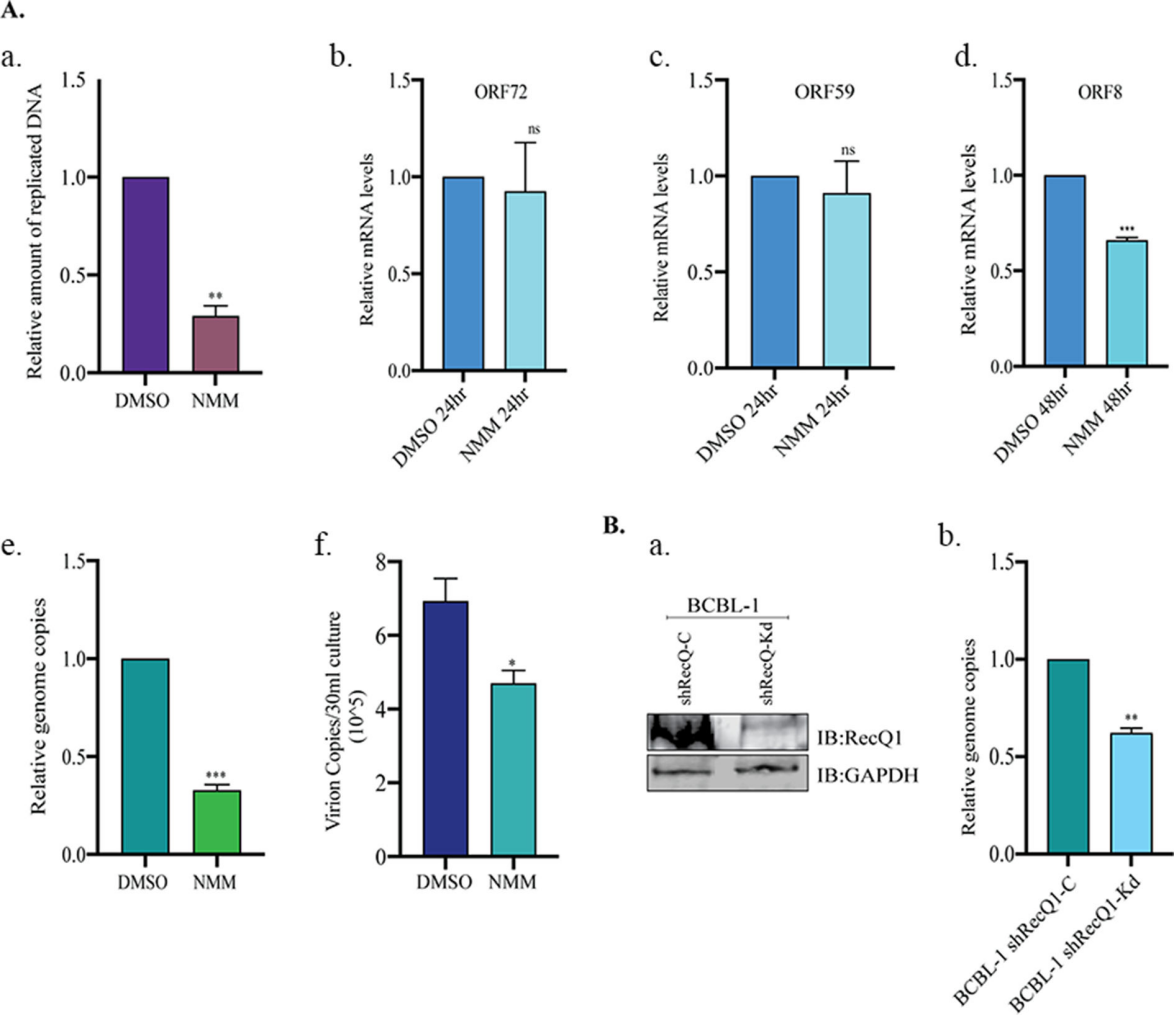
RecQ1 is essential for lytic DNA replication. (**A**) BCBL-1 cells were treated with DMSO or 2 mM NMM and induced for lytic reactivation (**a**) 24 hours post lytic induction; cells were labeled with IdU, genomic DNA was extracted, newly replicated DNA was pulled down anti-IdU antibody, and the DNA was quantified using primers specific for oriLyt region. (**b**) Twenty-four hours post lytic induction, mRNA was extracted, cDNA was synthesized and quantified using latent gene, ORF72 specific primers. (**c**) Twenty-four hours post lytic induction, mRNA was extracted, cDNA was synthesized and quantified using immediate early lytic gene, ORF59 specific primers. (**d**) Forty-eight hours post lytic induction, mRNA was extracted, and cDNA was synthesized and quantified using late lytic gene, ORF8 specific primers. (**e**) Twenty-four hours post lytic induction, genomic DNA was extracted, and viral genome copies were quantified using primers specific for genomic DNA sequence of RTA. (**f**) Ninety-six hours post lytic induction, the virus was concentrated from the supernatant of induced cells and quantified using ORF73 primers. (**B**) (**a**) Immunoblot showing depletion of RecQ1 in control (shRecQ1-C BCBL-1) and knockdown (shRecQ1-Kd BCBL-1) cells 24 hours post lytic induction. (**b**) shRecQ1-C BCBL-1 and shRecQ1-Kd BCBL-1 cells were induced for lytic reactivation for 24 hours, following which genomic DNA was extracted, and viral genome copies were quantified using primers specific for the genomic DNA sequence of RTA.

We investigated the involvement of RecQ1 in lytic DNA replication by using a pharmacologic inhibitor of RecQ1 unwinding activity by the stabilization of G4 sites as well as the depletion of RecQ1 protein levels through shRNA. *N*-methyl meso-porphyrin IX (NMM), a G4 DNA stabilizing ligand, selectively inhibits the unwinding of G-quadruplexes by the RecQ helicases with an inhibition constant, K_i_, of ~1.0 µM but not for dsDNA or Holliday junctions (K_i_ of ~25 µM (62). To this end, we treated the KSHV-infected cells with 2 µM of NMM for specifically blocking the RecQ-mediated unwinding of G4 sites and assaying the amount of DNA replicated following lytic reactivation. We achieved this by analyzing the amount of newly replicated DNA in NMM-treated cells following IdU labeling immunoprecipitating those newly synthesized DNA. The nascent DNA was pulled down using an anti-IdU antibody and was quantified using oriLyt-specific primers. Upon comparison, we observed that the amount of newly replicated DNA was reduced in cells treated with NMM compared to control DMSO-treated cells (Fig. 5A, panel a). Since RecQ1 inhibition was negatively affecting active lytic replication, we wanted to analyze the effect of RecQ1 inhibition on viral lytic gene expression and viral genome persistence. This was tested by quantifying the transcripts of representative genes of immediate-early and late genes, i.e., ORF59 and ORF8, respectively. NMM-treated cells showed a reduction in the transcription of late genes with no significant effect on early and latent genes (Fig. 5A, compare panels b through d). Upon observing the impact of NMM on late gene transcription, we asked whether that had any impact on viral genome copies. For this, we extracted genomic DNA and compared viral copies in DMSO or NMM-treated BCBL-1 cells induced for lytic reactivation. We found a reduction in the viral genome copies in cells treated with NMM as compared to the DMSO-treated cells (Fig. 5A, panel e). Having established the inhibitory effect of NMM on lytic DNA replication and viral genome copies, we wanted to test whether that impacted the production of cell-free virions. Not surprisingly, there was a decrease in the virions among cells treated with NMM compared to control cells (Fig. 5A, panel f).

Collectively, these findings led to the conclusion that the unwinding of the G4 site was essential for KSHV lytic DNA replication as its inhibition negatively affected the amounts of actively replicated DNA and the production of virions. Additionally, we tested the relevance of RecQ1 to lytic DNA replication through shRNA-based depletion of RecQ1. BCBL-1 cells, stably transduced with shRecQ1 lentivirus, were assayed for the depletion of RecQ1 post doxycycline treatment, which showed a significant reduction in RecQ1 levels (shRecQ1-Kd) as compared to control cells (shRecQ1-C) (Fig. 5B, panel a). Furthermore, BCBL-1 cells with a reduced RecQ1 level (shRecQ1-Kd) showed a significant reduction in the viral genome copies following lytic replication when compared to the control cells (shRecQ1-Kd BCBL-1), confirming the contribution of RecQ1 to the replication of viral genome during lytic replication (Fig. 5B, panel b). We confirmed that the depletion of RecQ1 itself did not trigger the reactivation of the lytic cycle demonstrated by the absence of immediate early/late genes.

### G4 disruption inhibited RecQ1 binding and lytic replication

**Fig 6:**
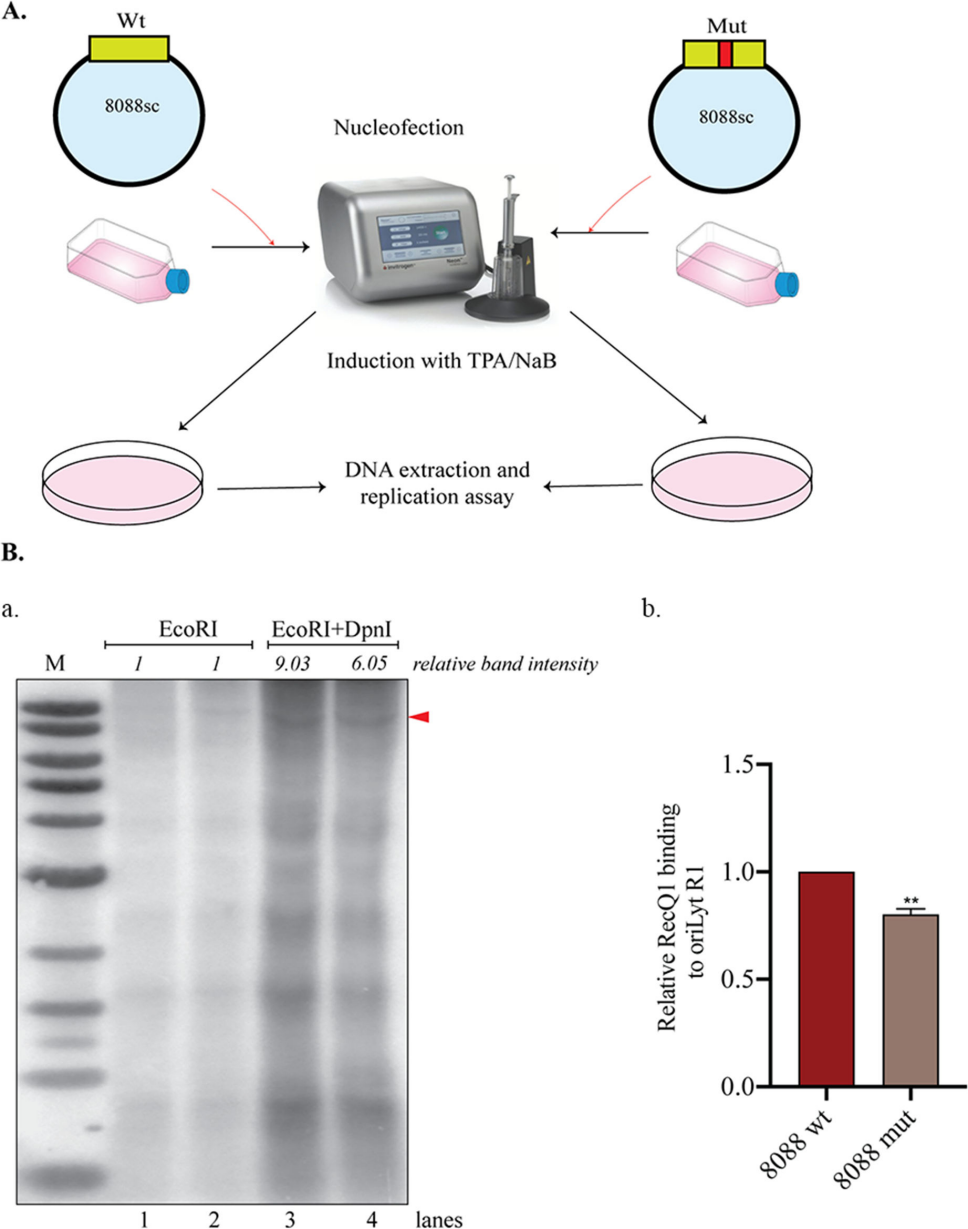
Disruption of G-quadruplex formation inhibited lytic replication and reduced RecQ1 binding to the G4 sites. (**A**) Schematic showing transfection of BCBL-1 cells with 8088wt or 8088mut plasmid. (**B**) BCBL-1 cells were transfected with 8088wt or 8088mut plasmid and induced for lytic reactivation. Forty-eight hours post-induction, total genomic was extracted using Hirt’s procedure and digested with either EcoRI or EcoRI and DpnI. DNA was subjected to Southern blotting and probing with an 8088sc specific probe. The band intensities were determined using ImageJ, and the relative band intensity was calculated using EcoRI lanes as 1 for respective plasmids, lanes 1 and 3 are 8088wt, and lanes 2 and 4 are 8088mut. M represents the marker lane. (**b**) ChIP assay was performed using 8088wt or mut transfected and 24 hours lytically induced BCBL-1 cells, where the cells were harvested, crosslinked, and chromatin-bound DNA was pulled down using RecQ1 antibody to determine the relative enrichment of RecQ1 at R1 region of oriLyt using specific primers.

The presence of G4 sites in oriLyt and the binding of RecQ1 to these sites prompted us to evaluate the functional relevance of these sites in the genome through their disruption. The G-quadruplex formation is highly dependent on the occurrence of four repeats of at least three G-residues separated by a few bases in between. We performed site-directed mutagenesis in the oriLyt plasmid (8088sc), which changed the G-rich sequence from ACGGGGTTGTTCGGTGGCGGGGGGGGGGGGG (8088wt) to ACGGGGTTGTTCGGTGGCAATAAGGGGGGGG (8088mut), which reduced the propensity of formation of G4 structures indicated by a drop in G-score. (Red fonts show G4 sites.) We tested these clones for their efficiency for DNA replication by performing a transient replication assay using these plasmids. A transient replication assay was performed by transfecting BCBL-1 cells with 8088wt or 8088mut plasmids, and 24 hours post-transfection, the cells were induced for lytic replication for 48 hours. Genomic DNA was extracted from these cells, followed by digestion with EcoRI to linearize or DpnI and EcoRI to determine the replicated copies after Southern hybridization. Upon quantifying the DpnI resistant/replicated DNA band, we observed a lower replication of the 8088mut plasmid than the 8088wt plasmid (Fig. 6B, panel a).

The above data showed RecQ1 to bind explicitly to the oriLyt G4 site compared to other regions in oriLyt. Since mutation of G4 sites deterred the formation of G4 structures (indicated by the G-scores), we wanted to test the effect of G-quadruplex disruption on RecQ1 binding. We achieved this by performing a chromatin immunoprecipitation assay on BCBL-1 cells transfected with 8088wt or 8088mut using RecQ1 antibody. BCBL-1 cells were transfected with 8088wt or 8088mut plasmids; and 24 hours post-transfection, cells were induced for lytic reactivation for additional 24 hours. ChIP was performed using the RecQ1 antibody, and the immunoprecipitated DNA was amplified using primers specific for R1. The relative binding of RecQ1 to R1 was reduced in cells transfected with 8088mut compared to cells transfected to the 8088wt, indicating that RecQ1 selectively binds to the G4 sites, which was compromised by mutation of the G4 sites (Fig. 5B, panel b).

### G-quadruplex stabilization impacted the initiation of lytic DNA replication

Since the G4 sites have been associated with replication initiation and a recent study established a link between G4 sites and origin activity in the mammalian genome, it prompted us to study the effect of these G-quadruplexes on lytic DNA replication origin of KSHV (61, 63). Herpesviruses are proposed to replicate via bidirectional theta-type replication and rolling circle replication, ultimately resulting in the generation of viral concatemers; however, the exact mechanism is not yet known (64). Here, we analyzed the effect of G-quadruplex stabilizing ligand (TMPyP4) on replication initiation through SMARD assay, a technique used to study replication events in EBV and KSHV (65, 66). TMPyP4 is another cationic porphyrin that interacts selectively with G-quadruplex structures and has been found to stabilize different types of G-quadruplexes, including those formed at the c-MYC promoter and human telomeric region (67 – 69). Although the nature and stoichiometry of TMPyP4 binding to the G-quadruplex have been controversial, its selectivity for the quadruplex relative to duplex DNA, investigated under molecular crowding conditions, proved to have enhanced selectivity for quadruplex DNA when compared to the duplex structure (70).

**Fig 7:**
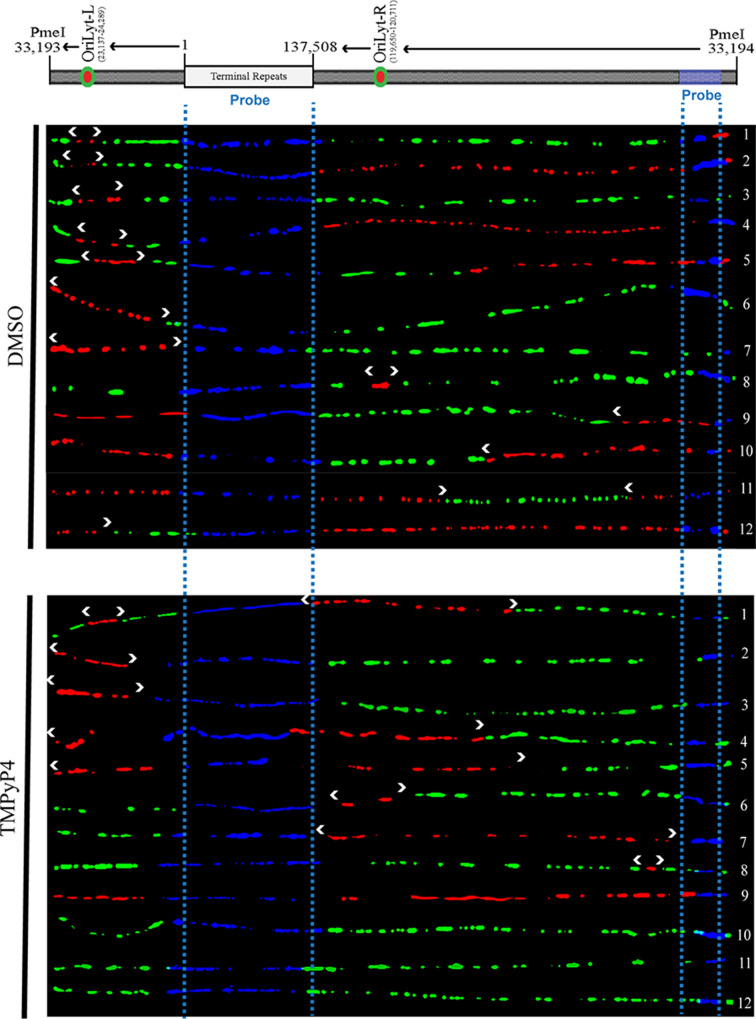
G-quadruplex stabilizing agent reduced initiation of lytic DNA replication from oriLyt. BCBL-1 cells were treated with DMSO or TMPyP4, induced lytic reactivation for 24 hours, and SMARD was performed on them. KSHV genome was detected by blue FISH probe signals. Red and green labels represented IdU (first label) and CldU (second label), respectively, following detection by immunostaining. White arrows denoted actively replicating DNA from the origin, i.e., oriLyt L, and green labels flanking red showed the bidirectional movement of the replication fork. The progressions of replication forks are marked with white arrows. Individual KSHV genome molecules (fibers) from the DMSO and TMPyP4-treated cells were analyzed for the number of molecules with distinct origins (red region flanked by green), which showed a reduced number of origins in the TMPyP4-treated set.

SMARD involves multiple steps beginning with sequential labeling of cells with halogenated nucleotides, IdU and CldU, embedding the cells in agarose plugs, lysis them, and digesting to linearize the viral genome. The plugs containing DNA were resolved on Pulse field Gel electrophoresis, where a band specific for the KSHV genome was excised, and DNA was extracted from the agarose plugs following GELase digestion. This was followed by stretching the DNA on positively charged glass slides and fluorescent *in-situ* hybridization (FISH) with biotinylated probes to identify and orient the individual viral genome. The labeled DNA was finally detected by monoclonal antibodies against IdU and CldU and fluorescently labeled secondary antibodies, whereas the biotinylated probes were detected by fluorescence-conjugated avidin. The DNA molecules used for determining the replication initiation sites possessed nucleotide analogs (IdU: red and CldU: green), which are arranged based on the increasing length of the IdU label. The molecules signifying a bidirectional initiation site show a progressively increasing IdU label (red signal) surrounded by the CldU label (green signal), indicating the replication fork’s bidirectional movement. Upon analysis of labeled DNA molecules from the BCBL-1 cells treated with DMSO or TMPyP4, we observed a reduction in the number of molecules with defined replication origin at oriLyt demonstrated by a relatively lower number of DNA molecules with red signal flanked by the green signal in the oriLyt region (Fig. 7). This supports the conclusion that stabilization of G-quadruplexes led to a reduction in lytic DNA replication.

### G-quadruplex stabilization inhibited lytic DNA replication

**Fig 8:**
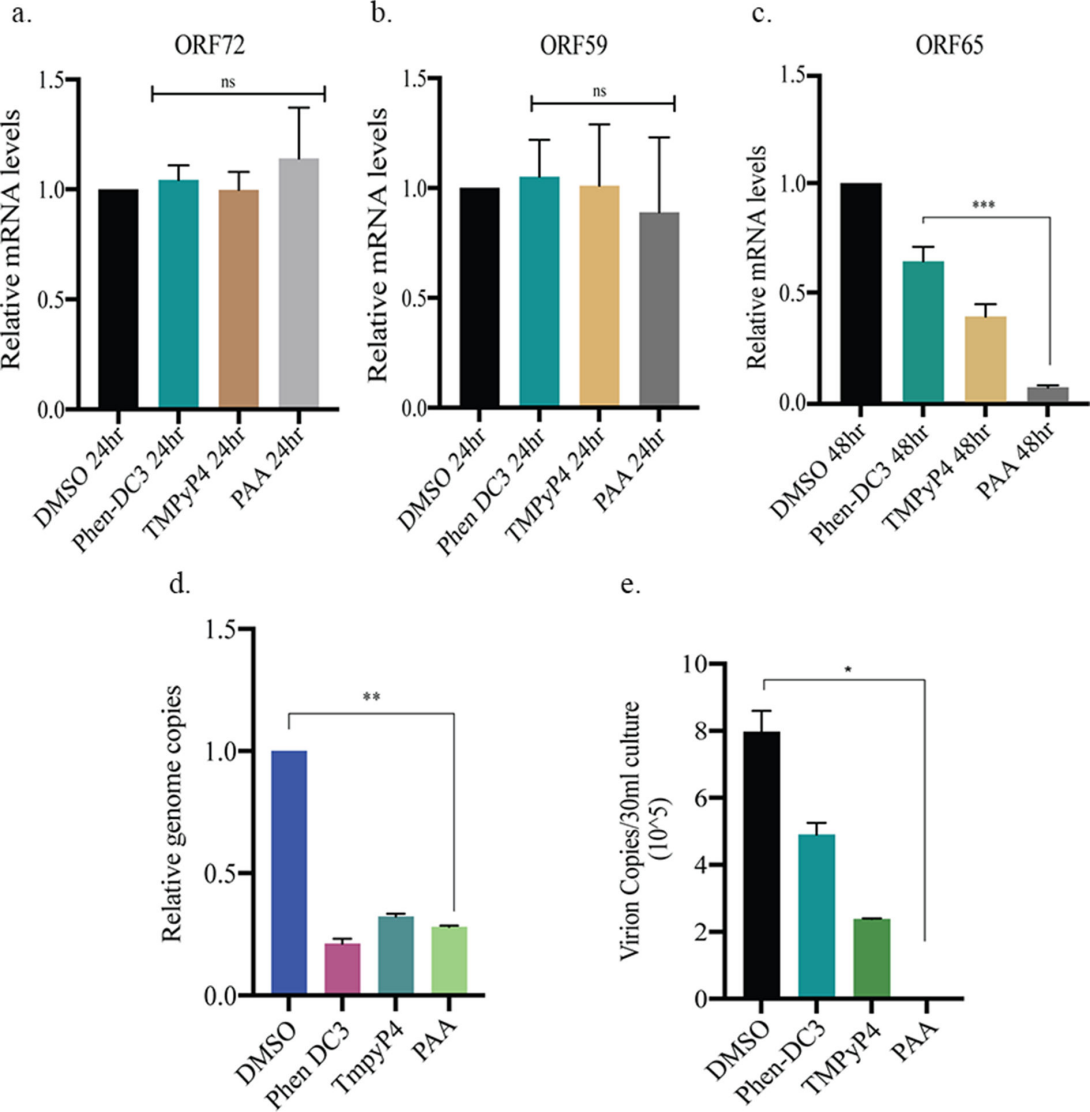
G-quadruplex stabilization inhibited lytic DNA replication. (**A**) BCBL-1 cells were treated with DMSO/10 mM Phen-DC3/10 mM TMPyP4/0.5 mM PAA and induced lytic reactivation. (**a**) Twenty-four hours post lytic induction, mRNA was extracted, cDNA was synthesized and quantified using latent gene, ORF72 specific primers (**b**) 24 hours post lytic induction, mRNA was extracted, cDNA was synthesized and quantified using immediate early lytic gene, ORF59 specific primers (**c**) 48 hours post lytic induction, mRNA was extracted, cDNA was synthesized and quantified using late lytic gene, ORF65 specific primers. (**d**) Twenty-four hours post lytic induction, genomic DNA was extracted, and viral genome copies were quantified using primers specific for the genomic DNA sequence of RTA. (**e**) Ninety-six hours post lytic induction, the virus was concentrated from the supernatant of induced cells and quantified using ORF73 primers.

Our results thus far showed that G4 sites regulate lytic DNA replication. To further evaluate the role of G4s in virus lytic replication and virion production, we used G-quadruplex stabilizing compounds to treat BCBL-1 cells and compared them with the control (DMSO) or a known inhibitor (phosphonoacetic acid; PAA) of lytic replication as a positive control. We used TMPyP4 as well as PhenDC-3, another G4 stabilizing agent, to confirm the impact of G4 stabilization on lytic replication. PhenDC3 has also been extensively studied as a G-quadruplex stabilizing agent in altering cellular and viral genome processes (71 – 73). We analyzed the effect of G-quadruplex stabilization through the detection of lytic genes as a reduction of lytic DNA replication impacts the transcription of lytic genes. This was tested by quantifying transcripts of immediate-early and late genes, i.e., ORF59 and ORF65, respectively. We found a significant reduction in the expression of late gene following the treatment of cells with G4 stabilizing compounds (PhenDC-3 and TMPyP4) and PAA compared to DMSO control, which did not show any significant effect on early gene and latent gene transcription (Fig. 8A, compare panels a through c). Next, we tested the effect of G4 stabilization on the viral genome copies in the host cell by quantifying the relative viral genome copies in BCBL-1 cells treated with DMSO or the G4 stabilizing compounds. Following the induction of lytic reactivation of the compound-treated cells, genomic DNA was isolated, and viral genome copies were quantified using gene-specific primers. Strikingly, we observed a reduction in viral genome copies in BCBL-1 cells treated with PhenDC-3 and TMPyP4 as well as in cells treated with PAA in comparison to the control DMSO-treated cells (Fig. 8A, panel d), leading us to conclude that stable G-quadruplex formation in the cells led to a defective genome persistence. Next, we assessed the effect of G4 stabilization on virion production by comparing the virion produced from the DMSO-treated cells to the cells treated with PhenDC-3, TMPyP4, or PAA. Our data demonstrated a significant reduction in cell-free virions in cells treated with the G4 stabilizing agents Fig. 8A, panel e). All these observations suggested that the formation of G4 structures in the KSHV genome inhibits lytic replication.

## DISCUSSION

KSHV, a member of the gamma herpesvirus family, maintains a biphasic life cycle consisting of latent and lytic cycles. The latency ensures a lifetime persistence of the virus in the host and the lytic DNA replication is essential to maintain viral reservoirs in infected cells, disseminate the virus, and promote tumorigenesis through the expression of oncogenic gene products (6). Latent DNA replication occurs synchronously with the cell cycle, and the duplicated genome is segregated into daughter cells. LANA is responsible for these functions in addition to latent DNA replication, where it recruits cellular DNA replication proteins to the origin of latent replication, oriP (74). Latency to the lytic reactivation switch can be triggered by various stimuli such as hypoxia, oxidative stress, viral coinfection, and chemicals (75). RTA is indispensable for lytic reactivation and is responsible for the switch of the viral life cycle from latency to lytic cycle through activation of its promoter and promoters of other viral genes, facilitates viral DNA replication through binding to the origin, acting as a component of the pre-replication complex and recruitment of cellular and viral factor to the origin, all of which are required for lytic DNA replication (14). This mode of replication differs from the latent mode in many ways as it originates from a distinct origin of replication, oriLyt, which requires the functioning of multiple viral proteins and results in the production of thousands of viral genomes. Apart from viral proteins, several cellular proteins have been shown to aid lytic DNA replication and bind to the oriLyt region, which includes topoisomerases (Topo) I and II, RecQ1, poly (ADP-ribose) polymerase I (PARP-1), DNA-PK and scaffold attachment factor A (SAF-A) to name a few (50).

G-quadruplexes are regulatory structures formed in G-rich DNA/RNA sequences that have been reported to play significant roles in biological processes. These structures have been identified in viral genomes such as HIV, EBV, HPV, HCMV, and KSHV (59, 76). Owing to the high GC content in their genomes, herpesviruses’ genomes could be expected to have a high propensity for forming G4 structures. Many studies have reported high putative quadruplex sequences (PQSs) in herpesvirus genomes (18, 59, 77). The regulation of DNA replication by G4 structures has been well studied, but their role in origin firing or replication initiation is relatively unexplored (61, 78, 79). A recent study provides strong evidence regarding the substantial role of G4s in the origin activity (63).

RecQ1, a cellular helicase, is a member of the RecQ helicase family, known for its roles in recombination, repair, and replication. In addition, other members of the RecQ family, such as BLM and WRN helicase, have been shown to unwind G4 structures and facilitate telomeric replication (61). RecQ1 has been shown to promote genome stability through its role in DNA damage response and facilitate restarting paused replication forks (80). Additionally, it has been proposed to have a role in origin activity and has been shown to participate in lytic DNA replication by binding to the oriLyt region of KSHV and EBV (50, 51, 79). We sought to explore the involvement of RecQ1 and the presence of G4s within the oriLyt, aiming to elucidate the significance of this association in the life cycle of KSHV.

Sequence analysis of oriLyt through the G4 predictive model revealed a strong possibility of the formation of G4 in a few regions, which was confirmed through CD spectroscopy and EMSA. The involvement of RecQ1 in the pre-replication complex and the viral replication centers through binding at oriLyt has shed light on the origin-based activity of RecQ1. G4 unwinding activity, another widely studied function of RecQ1, could explain its engagement at oriLyt. As an extension to previous studies of RecQ1 in KSHV, we found RecQ1 to be selectively enriched at the G4 forming site in oriLyt as compared to any other region, and this was confirmed through biotinylated DNA pull-downs and chromatin immunoprecipitation assays.

Moreover, the role of RTA in RecQ1-oriLyt binding was also substantiated by a reduced pull-down of RecQ1 at oriLyt in RTA-deleted cell lines. A reduction in genome copies of the virus in RecQ1-depleted cells showed further evidence of the role of RecQ1 in lytic replication. More interestingly, the inhibition of G-quadruplex unwinding by stabilization of G4 through the use of NMM led to a reduction in the amount of actively replicated DNA, genome copies, virion production, and late gene expression. Additionally, we demonstrated the importance of G-quadruplexes on the replication of oriLyt plasmid containing either wild-type or disrupted G-quadruplex sites, which showed inhibition in active lytic replication and a reduction in RecQ1 binding to the oriLyt region.

**Fig 9:**
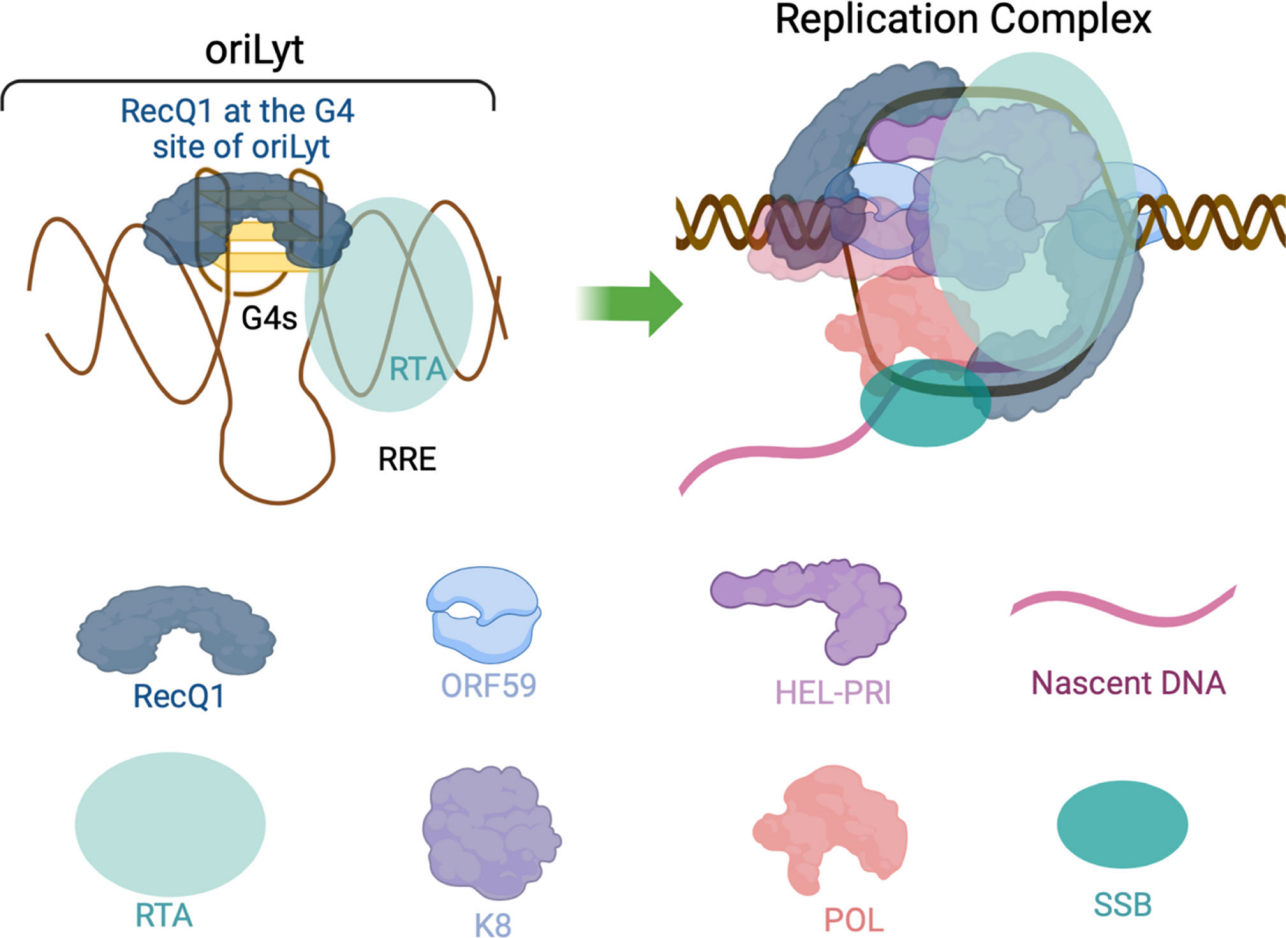
Proposed model of G-quadruplex mediated replication at KSHV oriLyt. (**A**) The RecQ1 (DNA helicase) binds to the G-quadruplex sites of the oriLyt and facilitates DNA replication, assisted by the binding of viral and cellular proteins. RRE, RTA response element, HEL-PRI: helicase-primase; POL, polymerase; SSB, single-stranded binding protein. Nascent DNA: newly replicated DNA; viral proteins, K8, ORF59, and RTA.

Additionally, we assessed the impact of G4 stabilization on lytic replication by employing G4-stabilizing compounds such as PhenDC-3 and TMPyP4. Importantly, the selectivity of TMPyP4 binding to the G-quadruplex has been controversial, but studies have shown that TMPyP4 displays enhanced selectivity for quadruplex DNA over duplex DNA under molecular crowding conditions, which may mimic the environment in living cells and promote the formation of G-quadruplexes (70, 81, 82). Detection of replication initiation on individual DNA molecules through SMARD revealed that cells treated with G4-stabilizing agents exhibited impaired initiation of replication from the origin. These observations were further substantiated in subsequent assays, wherein G4 stabilization led to a decrease in viral genome copies, diminished expression of late-lytic genes, and reduced production of progeny virions. Collectively, our findings emphasize the regulatory role of G-quadruplex formation in the oriLyt region in governing the initiation of lytic DNA replication of KSHV, with RecQ1 binding at the G4 sites potentially facilitating replication by unwinding these secondary structures (Fig. 9). Nevertheless, further investigations are required to elucidate the precise mechanism of how RecQ1 contributes to the replication at the origin of lytic DNA replication.
